# A laboratory and in-ward evaluation of cholesterol assays on the Ames Seralyzer

**DOI:** 10.1155/S1463924685000384

**Published:** 1985

**Authors:** Lesley M. Nelson, Robert S. Clark, Callum G. Fraser

**Affiliations:** 1 Department of Biochemical Medicine Ninewells Hospital and Medical School Scotland Dundee DD1 9SY UK; 2 Departments of Medicine Ninewells Hospital and Medical School Dundee DD1 9SY Scotland


**Journal of Automatic Chemistry**/*Journal of Clinical Laboratory Automation*, **Vol. 7, No. 4 (October–December 1985), pp. 173–176**

### A laboratory and in-ward evaluation of cholesterol assays on the Ames Seralyzer

**Lesley M. Nelson*, Robert S. Clark† and Callum G. Fraser***

*Departments of Biochemical Medicine* and Medicine†, Ninewells Hospital and Medical School, Dundee DD1 9SY, Scotland, UK*

### Introduction

Recently there has been renewed interest in the detection and treatment of hypercholesterolaemia; these being of particular importance in young and middle-aged patients with myocardial infarction [1]. It has lately been shown that, contrary to existing dogma, measurement of serum or plasma cholesterol levels within the first 24 h after infarction is a reliable way to assess prognosis or advise treatment [2]. Moreover, if results of cholesterol analyses were available without undue delay while the patient was still hospitalized in the short-stay coronary care unit, the treatment would be initiated by the specialist cardiologist and the required screening of first-degree relatives visiting the patient might be easier to motivate.

One approach to providing results in a timely manner is to perform analyses closer to the patient [3]. Although cholesterol analyses have been performed in an out-patient clinic [4], there does not appear to be data on the provision of such assays in a coronary care unit. The Ames Seralyzer is a system, based upon reflectance spectroscopy and use of solid-phase reagents, which has been stated to be convenient and reliable [5], although not without problems and difficulties [7 and 8]. Little data are available on the performance of cholesterol assays and the system was therefore subjected to a full evaluation in the laboratory under optimal conditions variance [9], and to a trial in the coronary care unit operated by three junior medical staff. Current criteria for acceptability of performance characteristics were applied to data assessment.

The Ames Seralyzer and reagent test strips were kindly provided by the manufacturer for the purposes of this study.

### Methods and results

### *The instrument*

The Ames Seralyzer is based upon reflectance spectroscopy and solid phase reagent strips, bar-coded for the analyte. Calibrators at two levels are supplied. Analysis requires separation of serum followed by a nine-fold aqueous dilution step and application of a measured aliquot (30 µl) to the strip. Enzymatic hydrolysis of cholesterol esters and oxidation of cholesterol produces peroxide which is measured by the oxidative coupling of 3-methyl-2-benzothiazolinone and primaquine disphosphate, in the presence of peroxidase, to produce a red-coloured complex. Incubation is timed automatically (135 s) and results are displayed digitally to one decimal place in mmol/l units.

### *Imprecision*

Within-run imprecision was assessed by analysis of three sera from patients, 20 times each, following calibration of the analyser. Between-run imprecision was assessed by analysis of thawed frozen portions of the same three sera 20 times in separate analytical runs; calibration was performed before each run, when a new bottle of test strips was used, or if CAL appeared on the instrument display panel. The results are shown in table 1.

### *Linearity*

A pool of patient's specimens with high levels of cholesterol was generated and two series of six samples made by dilution of aliquots of this pool with both water and portions of a pool of patient's specimens with low levels of cholesterol. All samples were analysed in duplicate in separate analytical runs. Using the criterion that the found value should lie within:

$$\text{the ideal} \pm \frac{2\,\text{SD between-run}}{\sqrt{\text{No. of replicates}}}$$

then the analyses were statistically linear up to at least 11.0 mmol/l.

### *Comparative inaccuracy*

100 Specimens from patients were analysed on the Seralyzer and on a Rotochem IIa parallel fast analyser (Aminco, Silver Spring, Massachusetts, USA), using CHOD/PAP enzymatic end-point colorimetric methodology standardized with Precilip EL material (both from Boehringer Mannheim, Mannheim, FR Germany). The relationship between the results is shown in figure 1; statistical analysis, as advocated by Westgard and Hunt [10], gave a correlation coefficient of 0·980 and the relationship:

$$\text{Seralyzer} = 0{\cdot}97 \text{ comparative method} + 0{\cdot}05.$$

The mean of the results obtained on the Seralyzer was 6·85 mmol/l and the mean by the comparative method was 7·00 mmol/l, which, by the Wilcoxon matched pairs signed rank test, was significantly higher ($p > 0{\cdot}001$).

*Address correspondence to Dr C. G. Fraser, Department of Biochemical Medicine, Ninewells Hospital and Medical School, Dundee DD1 9SY, Scotland, UK.*

*Table 1. Within-run and between run imprecision.*

| Level | Within-run | | | | Between-run | | | |
|---|---|---|---|---|---|---|---|---|
| | *N* | Mean (mmol/l) | SD (mmol/l) | CV(%) | *N* | Mean (mmol/l) | SD (mmol/l) | CV(%) |
| Low | 20 | 3·4 | 0·18 | 5·3 | 20 | 3·3 | 0·25 | 7·8 |
| Medium | 20 | 6·7 | 0·19 | 2·8 | 20 | 6·5 | 0·17 | 2·6 |
| High | 20 | 9·8 | 0·28 | 2·9 | 20 | 9·8 | 0·34 | 3·5 |

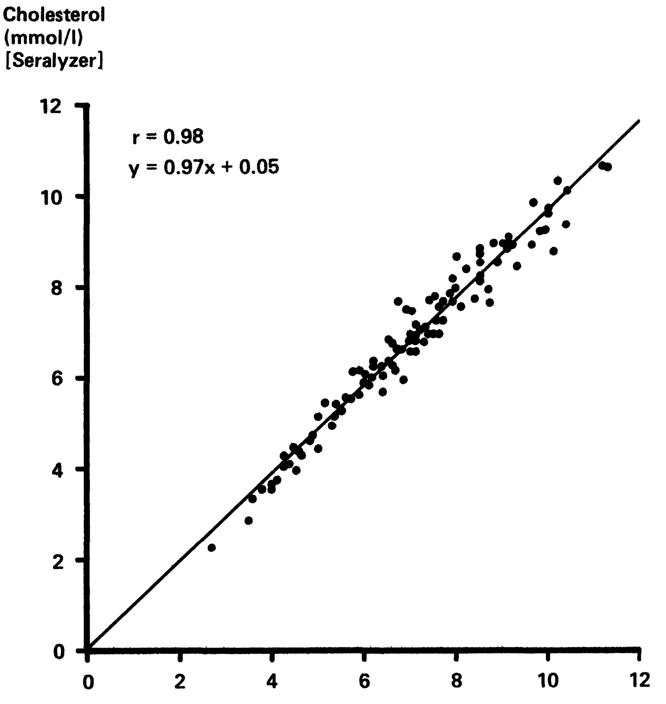

*Figure 1. Comparison of cholesterol results on 100 patients' specimens assayed by the Seralyzer in the laboratory and by the laboratory.*

*Table 2. Analysis of materials with assigned values.*

| Material | Assigned value (mmol/l) | Allowable error (mmol/l) | Value found (mmol/l) |
|---|---|---|---|
| 1 | 6·14 | ±0·19 | 6·50 |
| 2 | 2·99 | ±0·29 | 3·10 |
| 3 | 4·49 | ±0·19 | 4·50 |
| 4 | 8·17 | ±0·39 | 8·37 |
| 5 | 5·43 | ±0·19 | 5·70 |
| 6 | 2·73 | ±0·29 | 2·73 |
| 7 | 4·14 | ±0·19 | 4·17 |
| 8 | 6·23 | ±0·19 | 6·70 |
| 9 | 8·17 | ±0·39 | 8·53 |

*Analysis of materials with assigned values*

Three samples from an external quality assurance scheme (UK NEQUAS, Birmingham, UK), three samples assayed by a number of laboratories to assist a manufacturer to assign values to a product, and three samples with assigned values obtained from the Lipid Standardization Laboratory (Centers for Disease Control, Atlanta, Georgia, USA), were analysed in triplicate in separate analytical batches. The results are shown in table 2, together with the acceptable error, this being defined as:

$$\pm \frac{2\,\text{SD between-run}}{\sqrt{\text{No. of replicates}}}$$

*Interference*

The effect of lipaemia was investigated by duplicate analyses of six samples generated by mixing portions of a lipaemic specimen with aliquots of a non-lipaemic specimen. The effect of icterus was determined in a similar manner using an icteric and non-icteric specimen. The effect of haemolysis was investigated, firstly by duplicate analyses of six samples produced by mixing portions of a specimen to which a small amount of an erythrocyte haemolysate had been added to give a haemoglobin of 5.3 g/l with portions of another specimen and, secondly by duplicate analysis of aliquots of a series of five samples which were produced by addition of small amounts of dilutions of an erythrocyte haemolysate (haemoglobin content: 136 g/l) to aliquots of a pool of patients' specimens.

*In-ward assessment*

The Seralyzer was placed in the Coronary Care Unit, Ninewells Hospital and Medical School, for three months. During this time, junior medical staff were encouraged to collect specimens from patients, centrifuge these and analyse them in duplicate along with a quality control material supplied by the laboratory. Analysis of the specimens was then done by the laboratory in duplicate using the comparative method. The instrument was calibrated by a member of the laboratory staff and the recommendations made regarding tests outside the laboratory [11] were adhered to as far as possible. Three members of the junior medical staff participated in the study, each for a period of one month.

The imprecisions calculated from the analyses of quality control material and from duplicate analyses of patients' samples, and the correlations of the in-ward and comparative method results, are shown in table 3 for each of the three participants. Doctors A and B analysed all specimens in duplicate on a single dilution of serum, whereas doctor C analysed 12 specimens by preparing two separate dilutions of serum, and 13 specimens using a single dilution of each; the performance achieved by the two techniques was not statistically significantly different. The relationship between the pooled results from the

*Table 3. Performance characteristics achieved for in-ward analyses.*

| | Quality control material | | | | Patients' specimens | | | | Regression analysis | | |
|---|---|---|---|---|---|---|---|---|---|---|---|
| Junior doctor | *N* | Mean (mmol/l) | SD (mmol/l) | CV(%) | *N* | Mean (mmol/l) | SD (mmol/l) | CV(%) | *r* | Slope | Intercept |
| A | 7 | 10·0 | 0·58 | 5·8 | 12 | 5·9 | 0·32 | 5·5 | 0·67 | 0·79 | +0.92 |
| B | 16 | 9·7 | 0·57 | 5·9 | 13 | 6·1 | 0·24 | 4·0 | 0·63 | 0·72 | +1·15 |
| C | 8 | 9·8 | 0·51 | 5·3 | 25 | 6·3 | 0·32 | 5·1 | 0·89 | 1·28 | −2·23 |
| Overall | 31 | 9·8 | 0·57 | 5·8 | 50 | 6·1 | 0·30 | 4·9 | 0·79 | 1·05 | −0·80 |

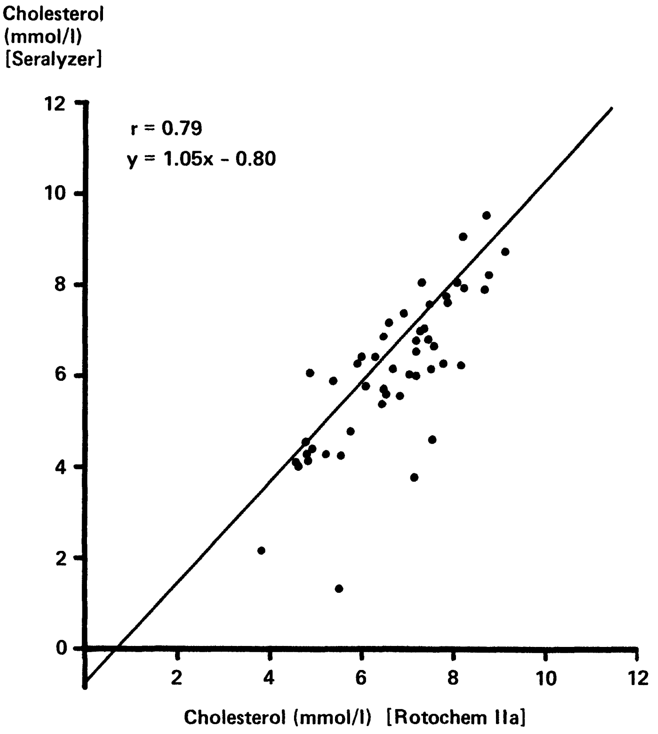

*Figure 2. Comparison of cholesterol results on 50 patients' specimens assayed by the Seralyzer operated by junior medical staff and by the laboratory.*

junior medical staff and the comparative method are shown in figure 2. Statistical analysis gave a correlation coefficient of 0.79 and the relationship:

Seralyzer = 1·05 comparative method −0·80.

The mean of the results obtained on the Seralyzer was 6·14 mmol/l, which was significantly less than 6·62 mmol/l for the comparative method ($p > 0{\cdot}03$: Wilcoxon matched pairs signed ranks). The performance achieved by junior medical staff was significantly worse than that obtained by laboratory both for duplicate analyses of the 50 patient specimens (F-test; $p > 0{\cdot}01$), the laboratory obtaining an SD of 0·09 mmol/l and a mean of 6·60 mmol/l, and for the analyses of quality control materials.

### Discussion

A previous limited laboratory-based evaluation of the Seralyzer for, *inter alia*, cholesterol assays stated that the system proved to be simple and easy to use, giving accurate and precise results [5]: these conclusions were simply based upon comparison with the method used in the laboratory of the authors.

Our findings suggest that, since the within-run and between-run imprecisions are not statistically different, the instrument is robust under laboratory conditions. The imprecision is not satisfactory, however, in that the currently accepted analytical goal for cholesterol analyses, based upon biological variation, is that the CV should be equal to, or less than, 2.4% [12]. Moreover, the World Health Organization has laid down criteria for use of cholesterol analyses in epidemiological studies [13], and the acceptability criteria of SD ≤0·17 at 2·6 mmol/l, SD ≤0·19 at 5·2 mmol/l, SD ≤0·21 at 7·8 mmol/l and SD ≤0·25 at 10·3 mmol/l are not fulfilled by the Seralyzer even under optimal conditions variance. We cannot envisage that usual methods for improvement of performance [14] could lead to the performance characteristics meeting analytical goals for imprecision.

The correlation between the results obtained with the Seralyzer and the in-house comparative method was good, but the results obtained were significantly different. Moreover, the analyses of materials with assigned values showed that the Seralyzer gave high results at values around 6·5 mmol/l. Since the treatment of hypercholesterolaemia is based upon comparison of laboratory results with preset numerical values, we believe that analyses must, in such circumstances, be without bias [15]. The WHO criterion [13] is that analytical bias should not be more than ±5.0%; this was not fulfilled at cholesterol levels of 6·14 and 6·23 mmol/l.

The Seralyzer had linearity which was satisfactory since the published goal is that analyses should be linear up to 10·3 mmol/l[12]. There was insignificant interference from lipaemia or icterus, but haemolysis caused significant lowering of result (0·5 mmol/l per g/l haemoglobin).

The performance achieved by the junior doctors was unsatisfactory. Comparison of the imprecision achieved in the ward for duplicate analyses with that obtained by the laboratory showed (F-test) that the laboratory was significantly better. Moreover, the correlations between the results obtained in the ward and by the comparative method were poor. These results confirm earlier findings regarding the use of both the Seralyzer by non-laboratory staff and other analytical equipment used in ward and clinics [16].

It is concluded therefore that the Ames Seralyzer is not an acceptable instrument for performance of cholesterol assays in either laboratory or ward. Clinical needs of phlebotomy within 24 h of admission, and return of result before patient discharge from coronary care units, must be fulfilled by adoption of other strategies.

Although the Seralyzer cannot be considered an acceptable method for cholesterol assays in the laboratory or ward it is possible that it has a role in identifying patients requiring follow-up. The laboratory performance in this study indicated that the accuracy of the results was acceptable at around 8 mmol/l although levels around 6 mmol/l were overestimated. The statistical postulates of Harris suggest that goals for imprecision can perhaps be less stringent for first line screening [17]. This goal (CV $\leqslant$10%) is fulfilled by the Seralyzer.

With adequate training, a dedicated nurse in a health centre might be able to achieve performance equivalent to that of the laboratory. Patients with cholesterol levels greater than 7·0 mmol/l, who were thus identified, could then have their cholesterol level more formally assayed and treatment, based upon one or preferably two laboratory derived cholesterol value(s), initiated.

### Acknowledgements

We thank Ames Co. for providing the Seralyzer and the reagents for this study and the junior medical staff for their participation.

### References

1. Oliver, M. F., *British Medical Journal*, **289** (1984), 1641.
2. Ryder, R. E. J., Hayes, T. M., Mulligan, I. P., Kingswood, J. C., Williams, S. and Owens, D. R., *British Medical Journal*, **289** (1984), 1651.
3. Marks, V., *British Medical Journal*, **286** (1983), 1166.
4. Fraser, C. G., Peake, M. J. and Calvert, G. D., *Medical Journal of Australia*, **1** (1979), 465.
5. Stevens, J. F. and Newall, R. G., *Journal of Clinical Pathology*, **36** (1983), 9.
6. Stevens, J. F., Tsang, W. and Newall, R. G., *Journal of Clinical Pathology*, **36** (1983), 598.
7. Clark, P. M. S. and Broughton, P. M. G., *Annals of Clinical Biochemistry*, **20** (1983) 208.
8. Clark, P. M. S. and Broughton, P. H. G., *Journal of Automatic Chemistry*, **5** (1983), 22.
9. Whitehead, T. P., *Advances in Clinical Chemistry*, **19** (1977), 175.
10. Westgard, J. O. and Hunt, M. R., *Clinical Chemistry*, **19** (1973), 49.
11. Anderson, J. R., Linsell, W. D. and Mitchell, F. L., *British Medical Journal*, **282** (1981), 743.
12. Elevitch, F. R., *Proceedings of the 1976 Aspen Conferences on Analytical Goals in Clinical Chemistry* (CAP), (1977), 4.
13. World Health Organization, *Standardization of Lipid Measurements* (WHO, 1983), 19.
14. Fraser, C. G., *Clinical Biochemist Reviews*, **3** (1982), 23.
15. Fraser, C. G., *Advances in Clinical Chemistry*, **23** (1983), 299.
16. Fraser, C. G. and Watkinson, L. R., in *Clinical Biochemistry Nearer the Patient*, Eds. Marks, V and Alberti, K. G. M. M. (Churchill Livingstone, London, 1985), 11.
17. Harris, E. K., *American Journal of Clinical Pathology*, **72** (1979), 374.

### ANALYTICA 86

*3 to 6 June 1986 at the Munich Trade Fair Centre*

The scientific programme for this international meeting is divided into symposia, posters and the 'Analytica – Forum München' (in this latter sector, exhibiting companies will present papers on developments in industrial research). Topics to be covered include:

Separation methods
- Chromatographic methods, especially TLC/HPTLC, GC, LC/HPLC
- Electrophoretic methods

Combined methods: MS-MS, HPLC-MS, GC-MS, GC-IR, LC-MS, LC-NMR, FT-GC, GC-FTIR
Emission spectroscopy
Bioluminescence, chemiluminescence, fluorimetry
NMR, its application *in vivo*
Radiochemical procedures
Topochemical procedures
Enzymatic analysis
Cell and organ culture based analysis
Dry support reagents including stick tests
Progress in development of reference methods.

*Enquiries about the commercial exhibition and registration to Münchener Messe- und Ausstellungsgesellschaft mbH, Analytica 86, Postfach 12 10 09, D 8000 München 12, FR Germany. Information about scientific contributions from Professor Dr H. Feldmann, Inst. für Physiologische Chemie der Universität, Goethestrasse 33, D 8000 München 2, FR Germany.*

